# Structural Basis for (2*R*,3*R*)-Taxifolin Binding and Reaction Products to the Bacterial Chalcone Isomerase of *Eubacterium ramulus*

**DOI:** 10.3390/molecules27227909

**Published:** 2022-11-16

**Authors:** Gottfried J. Palm, Maren Thomsen, Leona Berndt, Winfried Hinrichs

**Affiliations:** Institute of Biochemistry, University of Greifswald, Felix-Hausdorff-Str. 4, 17489 Greifswald, Germany

**Keywords:** flavonoid, crystal structure, substrate recognition, Michael addition, chalcone, taxifolin, alphitonin, gut microbiota

## Abstract

The bacterial chalcone isomerase (CHI) from *Eubacterium ramulus* catalyses the first step in a flavanone-degradation pathway by a reverse Michael addition. The overall fold and the constitution of the active site of the enzyme completely differ from the well-characterised chalcone isomerase of plants. For (+)-taxifolin, CHI catalyses the intramolecular ring contraction to alphitonin. In this study, Fwe perform crystal structure analyses of CHI and its active site mutant His33Ala in the presence of the substrate taxifolin at 2.15 and 2.8 Å resolution, respectively. The inactive enzyme binds the substrate (+)-taxifolin as well defined, whereas the electron density maps of the native CHI show a superposition of substrate, product alphitonin, and most probably also the reaction intermediate taxifolin chalcone. Evidently, His33 mediates the stereospecific acid-base reaction by abstracting a proton from the flavonoid scaffold. The stereospecificity of the product is discussed.

## 1. Introduction

Flavonoids are polyphenolic compounds representing a large class of secondary metabolites produced by plants. These compounds are responsible for flower colouration to attract pollinators. They are well known for their antioxidative abilities and are antimicrobial phytoalexins. In addition to other properties, they are also specific bacterial regulators governing the expression of *Rhizobium* genes involved in root nodulation [1,2]. Potential pharmacological properties, such as anti-infectivity, anti-inflammatory, anti-tumor, and immune response regulation, are evident [3].

The enzymatic activity of chalcone isomerases independently evolves twice. One type of these bio-catalysts is required in the biosynthesis of flavonoids in plants, but are not exclusively unique to plants [4]. The reverse biological function, flavonoid degradation, has been described for another type, first observed in the gut bacterium *Eubacterium ramulus* [5], and recently in other bacteria [6].

In the course of flavonoid biosynthesis in plants, the chalcone isomerase from *Medicago sativa* catalyses stereospecific intramolecular cyclization of 4,2′,4′,6′-tetrahydroxychalcone (the naringenin chalcone) by an oxa-Michael addition to (2*S*)-naringenin [7]. This skeleton can be further modified to various bioactive flavonoids. The evolutionary history of the plant chalcone isomerase [8], the three-dimensional structure [9], and the reaction mechanisms have been investigated in detail [10,11].

The reverse reaction by ring opening of (2*S*)-naringenin forming the naringenin chalcone has been utilized as a first step in a bacterial flavanone-degradation pathway. A flavonoid-degrading gut bacterium was first observed by Schneider et al. [12] and later identified as *Eubacterium ramulus* [5,13].

The naringenin catabolic pathway is a multi-step enzymatic degradation cascade, but in vitro the bacterial CHI catalyses more efficiently the energetically favourable ring closure of the chalcone, comparable to the flavanone-synthesis step in plants [14]. Thus, efficient degradation of flavanones can only occur in a steady-state equilibrium when the chalcone is subsequently eliminated. The enzymes involved in step-by-step naringenin degradation have been characterised as a chalcone isomerase CHI [14], an enoate reductase ERED [15], and a phloretin hydrolase [16]. The biocatalytic conversion of naringenin to phloretin was shown using recombinant CHI and ERED expressed in *E. coli* [15] with the aim of biotechnological applications.

A PSI-BLAST [17] search of sequence databases revealed no identity of *E. ramulus* CHI compared with plant CHI or other characterised polypeptide sequences (<10%). Thus, neither structural homology nor a close evolutionary relationship was expected and, recently, we determined the crystal structures of the bacterial CHI from *E. ramulus* without substrate and in complex with (2*S*)-naringenin at 1.8 and 2.0 Å resolution, respectively [18]. The quaternary structure is a hexamer with D_3_ symmetry (trimer of dimers). The tertiary structure of bacterial CHI (polypeptide with 282 amino-acid residues) is separated into a catalytic- and solvent-exposed domain, both with ferredoxin-like folds, completely different to that of the plant CHI, suggesting that the enzyme activities convergently evolved from different ancestor proteins. The two-domain structure of the bacterial CHI is closely related to a chlorite dismutase and the stress-related protein SP1, despite the lack of any functional relationship.

The substrate-binding site of plant CHI is solvent-exposed during the catalytic process, but the bacterial CHI has a lid structure (amino-acid residues 106–130) closing the active site upon substrate binding. This lid structure is part of the catalytic domain and was observed in different conformational states (open/closed) with respect to the active site. The shielding lid is unambiguously the main reason for the 19-fold higher efficiency of the bacterial CHI [18].

The bacterial CHI is only related to the plant CHI with respect to the products of the catalysed oxa-Michael addition. The general acid–base mechanism relies on the His33 side chain in bacterial CHI, in contrast to the catalytic water molecule in plant CHI [10].

**Figure 1:**
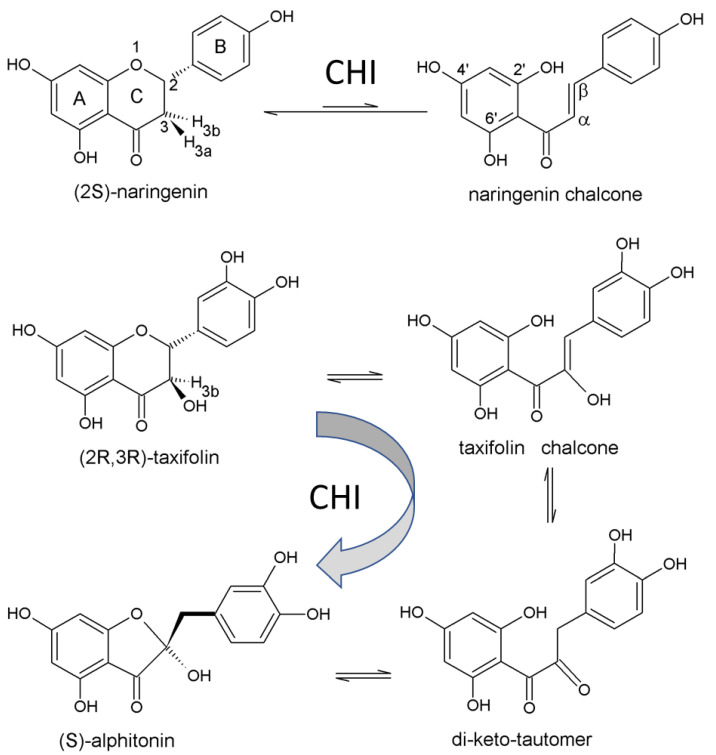
Molecular structures of the discussed compounds. The common atom numbering and the ring labels of the C6-C3-C6 framework are given. On top is the substrate/product equilibrium (2*S*)-naringenin/chalcone. The reversible oxa-Michael addition is catalysed by His33 by abstracting the proton H3b. Note: The in vitro product is (2*S*)-naringenin. CHI converts (2*R*,3*R*)-taxifolin to alphitonin. Again, the H3b proton is abstracted by His33. The chalcone and its di-keto tautomer are proposed reaction intermediates which enable ring contraction [20].

The imidazole ring of His33 induces the ring opening of (2*S*)-naringenin to form naringenin chalcone by proton abstraction at C3 of the C ring of the flavanone moiety (Figure 1). The stereochemistry of the isomerase reaction mechanism has been verified by mutagenesis studies and specific ^1^H/^2^H exchange observed by NMR experiments [18].

Chemical and enzymatic conversion of taxifolin to alphitonin has been described [19]. Taxifolin processing has been reported [20], taking into account our previous crystallographic and enzymological characterization of CHI [18]. Further, (2*R*,3*R*)-Taxifolin shares its scaffold with (2*S*)-naringenin and the initial reaction step of abstracting a proton at C3, but the additional hydroxyl group at C3 initiates further reaction steps determining different products (Figure 1). The opposite enantiomers of the substrates have been characterised as competitive inhibitors [20].

In this study, we underline the catalytic activity of the imidazole of His33 by comparison of wild-type CHI and its inactive His33Ala variant by crystal structure analyses in presence of taxifolin. In this context, the bacterial CHI is of general interest for biotechnological applications in stereospecific flavonoid syntheses and flavanone conversion.

## 2. Results

### 2.1. Crystal Structures

The protein preparation, including cloning, expression, purification, and crystallisation of CHI and the His33Ala variant (CHI_H33A), followed our previous protocols [18]. Crystals of native CHI and CHI_H33A were soaked in the substrate (2*R*,3*R*)-dihydroquercetin ((+)-taxifolin, Protein Data Bank (PDB) three-letter code DQH) and X-ray diffraction data were collected. The natural occurring (+)-enantiomer was used because its stereoisomer was characterised as a competitive inhibitor [20]. We obtained crystal structures of native CHI and two different structures of CHI_H33A at 2.15 Å and 2.8/3.0 Å resolution, respectively. Crystallographic analyses were performed by molecular replacement and subsequent model building and refinement. Statistics of data collection and structure refinement are summarised in Table A1 and Table A2, respectively.

### 2.2. Common Structural Features of the Proteins

First, common structural features of the three structure analyses are described, followed by the observed differences in substrate and product binding to the active site. All active sites are occupied by substrate DQH and/or product molecules due to artificially high concentrations used for soaking.

The native CHI crystallised with one hexamer in the asymmetric unit isomorphous of our previously reported crystal structures (PDB entries 4C9S, 4C9T, 4D06). This quaternary structure is also observed for CHI_H33A. The striking difference is that the hexamers are slightly displaced by about a 10 Å shift along the internal threefold axis. As a consequence, three adjacent hexamers form an extended asymmetric unit along the crystallographic *c*-axis, which is accordingly almost three times longer. The crystal packing of both unit cells is almost congruent with the hexamer as packing unit and the orthorhombic space group *I*2_1_2_1_2_1_ is preserved. The higher ammonium sulphate concentration in the crystallisation buffer for CHI (1.8 M) compared to CHI_H33A (1.5 M) is obviously the reason for the modified unit cell and a 3.4% shrinkage in volume per monomer of the native CHI.

In these structures, the quaternary assembly and the tertiary structures of all CHI protomers are conserved. Previously, the lid structure had been observed in different states (open/closed) with respect to the entry tunnel of the active site. In the (2S)-naringenin complex structure (PDB entry 4D06), the lid was observed in the closed conformation in three of the six subunits with bound product, but also in open conformation in subunits with occupied active sites. Here, the lid is always in the open conformation; i.e., no electron density has been observed for the peptide residues 106–130. In all crystal structures of CHI and variants, there is sufficient space in the crystal packing to allow conformational changes of the lid.

The lid structure is the only part of the enzyme to have functional mobility. A superposition of the hexamers of the CHI/naringenin complex (PDB entry 4D06) with our new crystal structures of wild-type CHI and the H33A variant reveal an RMSD on Cα atoms of <0.4 Å. Thus, the polypeptides of CHI form a remarkable rigid framework on the tertiary and even quaternary structure level.

### 2.3. The Taxifolin Binding to CHI_H33A

**Figure 2:**
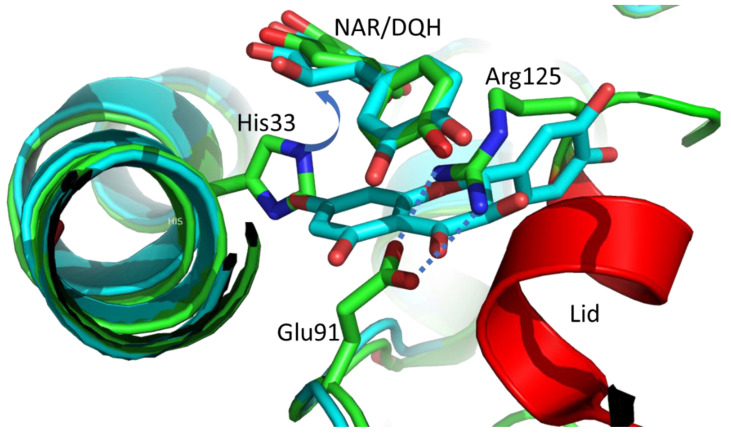
The figure shows a superimposition of the NAR complex of wild-type CHI (PDB entry 4D06) with CHI_H33A with two DQH molecules (C atoms coloured light blue) in and at the active site (protomer A, light blue). The labelled NAR/DQH are in the catalytically relevant position. The general acid–base mechanism relies on the His33 side chain abstracting the H3b proton at C3 of NAR or DQH (purple arrow). The NAR complex has the closed lid conformation (red α-helix, green C atoms). The second DQH of CHI_H33A clashes in the wild-type CHI with His33 imidazole (A ring), the short α-helix (red) of the closed lid, and crosses the salt bridge of Glu91–Arg125 (C ring). All O and N atoms are in red and blue, respectively.

The diffraction data of two CHI_H33A crystals soaked with DQH reveal electron-density maps identifying distinct substrate binding in the active site and the entry tunnel. The inactive enzyme binds the substrate in an identical position, as found for NAR (Figure 2). The superposition reveals a root-mean-square deviation (RMSD) less than 1 Å for DQH and NAR, and all substrate-recognizing side chains remain in the same position. Thus, the hydrogen atom at C3 is in the same position as previously described for catalysis [18].

The C-ring of a second DQH forms stacking interactions (3.5–4.0 Å) to the B-ring of the substrate-like positioned DQH. The second DQH points with its A-ring to the position of the truncated imidazole side chain 33. The B-ring adopts the position of a helical turn (residues 121–125) of the closed lid observed in the NAR complex, and the C-ring runs through the centre of the salt bridge Glu91–Arg125 (Figure 2). This artificial and less specific DQH binding is in competition with lid closure. This is in line with an already discussed low energy barrier for conformational changes for lid closure [18].

**Figure 3:**
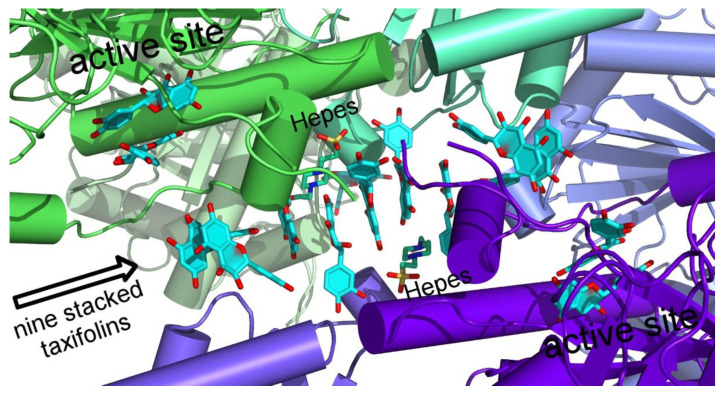
Nine DQH molecules form a stack in a horizontal oriented cleft between two CHI hexamers. Both ends are close to DQH dimers of active sites on the top left and bottom right. DQH molecules are represented as stick models with closed ring planes (oxygen red, carbon light blue). Two HEPES buffer molecules in the cleft are shown as stick models (C, N, and S atoms coloured green, blue, and yellow, respectively). CHI monomers with different colours represented by α-helices (cylinders), β-strands (arrows), and connecting loops. In the centre the open ends are due to the not observed lid structure.

One data set (CHI_H33A-overload) shows an overload with substrate molecules additionally binding in a cleft between two hexamers (see Figure 3). Five DQH molecules are stacked with their A,C-plane, flanked by two HEPES buffer molecules. On both ends of this stack, the π-system of the A,C plane accepts hydrogen bonds from the B-ring hydroxyl groups of another DQH, whose B-ring rings are perpendicular to the stack. Both terminal DQH molecules are accompanied by another DQH in the orientation as described for the corresponding pair in the active site. Both DQH pairs adopt the position of the closed conformation of the lid (amino-acid residues 111–116 in PDB entry 4D06). Both ends of the DQH assembly are next to active site DQH pairs, but without direct contact (minimum distances 7–10 Å).

### 2.4. The Taxifolin Binding to Native CHI

In a previously reported crystal structure of CHI (PDB entry 4D06), NAR, or alternatively, the naringenin chalcone, are observed in different active sites, but with distinct electron density. The electron-density maps of native CHI soaked with DQH show a complicated disorder in the active sites. Clearly, this structure analysis is not the doubtless identification of a distinct molecule in the active site, but more of a snapshot of the enzyme at work.

**Figure 4:**
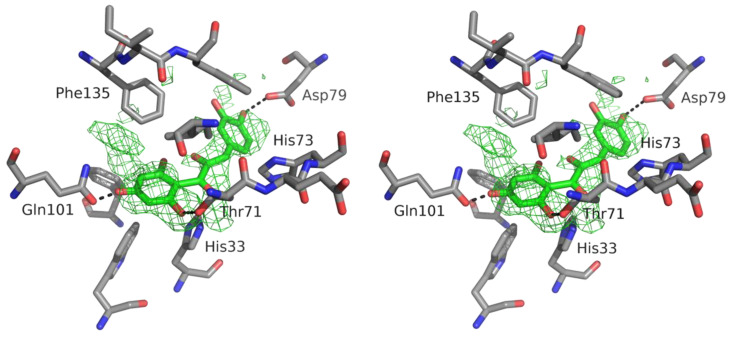
Stereo view with electron density map of active site CHI with bound reaction intermediate (green) representative of the mixture of reaction species. The Fo-Fc omit map (3 σ, green) was calculated without substrate/product molecules. The hydrogen bonds to the taxifolin chalcone are shown as dashed lines.

The C6-C3-C6 framework cannot be clearly identified as one of the possible substrate or product molecules. We see two electron density bubbles at the positions of the A and B ring, but a gap for the connection (Figure 4). This can be interpreted as a superposition of substrate, intermediates, and product caused by equilibria on the reaction pathway.

For both substrate molecules, NAR and DQH, the reaction product is a chalcone, but the α-hydroxy substituent at C3 makes the difference. The DQH chalcone is in equilibrium with a di-keto-tautomer and the reaction proceeds to alphitonin [20]. The observed electron density might be caused by alternative substrate and product binding in the active sites, but additionally, the reaction intermediates, the chalcone and the di-keto-tautomer, should be considered.

In the course of the enzymatic process, the C ring atoms C2, C3 change their configuration twice (sp^2^/sp^3^, see Figure 1) and undergo the most prominent movements. This causes discontinuous electron density. The aromatic A and B rings are likely positioned by hydrogen bonds, but slight plane rotation and translation are forced by the central rearrangement and weaken the electron density.

Initial crystallographic refinement was tested with each of the four molecules of the reaction pathway. According to refinement statistics and electron density maps, the final model building and refinement was performed with the taxifolin chalcone, which marginally turned out as the best. Refinement with superposition of possible ligands with coupled occupancies makes no sense because of the lack of electron density for the A,B ring connection.

No additional substrate binding in the active site was observed. This is reasonable, because it would interfere with lid closure (as outlined above for CHI_H33A), inhibit enzymatic catalysis, and thus, prevent the observed mixture of reaction path species.

### 2.5. Minor Comments on CHI Folding

We observed deviations from planarity for the peptide bonds 270/271 (ω = 208°) and 273/274 (ω = 205°). These are large, but not unseen [21,22]. The distortion is conserved in all measured monomers. Another intrinsic property of the fold is the cis-peptide bond at Pro250, which we observe to be conserved as a rare non-proline cis-peptide bond in the crystal structure of the Pro250Ala variant (PDB entry 4D4F).

## 3. Discussion

Our structural results are in line with and support previous knowledge of the catalytic function of CHI based on crystal structures, enzyme kinetics of wild type and various mutants, mechanistic studies with 1H-NMR measurements, and standard biophysical protein characterization [15,18,20].

**Figure 5:**
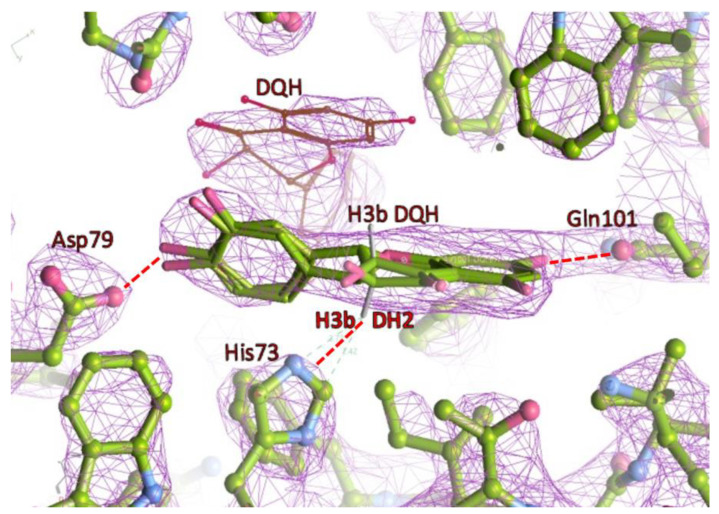
Superposition of DQH with DH2 fitted to the final electron density (2Fo–Fc map; 2.0 σ contour level) of the CHI_H33A-taxifolin structure. Note the antiparallel direction of the C3–H3b orientation. The H3b of DH2 is at a distance of 2.4–2.7 Å to imidazole ring atoms (red dashed line). The second DQH (line representation) occupies the position of the missing imidazole of His33. Hydrogen bonds of Asp79 and Gln101 are shown for orientation (dashed lines).

We used the (+)-taxifolin (DQH) for crystal soaking because the (-)-enantiomer (PDB three-letter code DH2) was characterised as a competitive inhibitor [20]. In the active site, the imidazole side chains of His33 and His73 are next to the substrate, but only His33 is the active residue. The superposition of DQH with DH2 allows the same positions of the A and B rings. The DH2 would also fit into the final electron density maps of the CHI_H33A structure, but enantiopure (+)-taxifolin was added during soaking and only DQH was present in the crystals. Due to C ring puckering, the C3 atoms and the attached hydroxyl oxygens of the stereoisomers are at a distance of ~0.6 Å and ~0.5Å, respectively. Their C-H vectors are anti-parallel and both point towards a histidine side chain. The Nε3 of the His73 imidazole is at a distance to C3 of DH2 suitable to discuss proton abstraction (~3.3Å) (Figure 5).

Why is His73 not catalytically active? His73 has no neighbours to polarize the imidazole ring, whereas Nδ1 of His33 imidazole is hydrogen bonded via a single water molecule to the carboxylate of Asp36.

The stereoselectivity of the enzymatically obtained alphitonin could not be resolved due to the fast racemization of alphitonin [19]. We propose (*S*)-alphitonin as an enzymatic product regulated by the lining of the active site. The hydroxyl O1 binds to C3 producing the five-membered ring closure of alphitonin. In Figure 4, the reaction intermediate (chalcone or di-keto tautomer) is shown in the active site. The oxygen atom at C3 (a hydroxyl or carbonyl oxygen) forms the hydroxyl of alphitonin, determining the stereoselectivity. The R-enantiomer becomes possible only if O3 moves into the direction of a hydrophobic cluster of side chains Ile12, Phe135, and Phe137. In this case, the estimated distance of the hydroxyl O3 to the methyl group (Cδ1) of Ile12 will be less than 3 Å. Most likely, O3 is guided into the opposite direction to His33 to form the S-enantiomer.

Another different way to obtain the (*R*,*S*)-alphitonin could be that the chalcone or its di-keto tautomer is released and ring closure takes place without stereochemical regulation.

The frequently used term “dual reaction” for CHI is misleading and should be avoided. The initial reaction step in flavonoid degradation is the proton abstraction of H3b by the imidazole of His33. In both, the flavanones (e.g., NAR) and the flavanonols (e.g., DQH), the first reaction step results in the corresponding chalcone. At this point, the 3-hydroxy substituent makes the difference, because it activates the C3 atom in the chalcone to allow further reaction, ending with the C-ring contraction, forming an auronol (e.g., alphitonin).

In both His33Ala structures, the additional DQH binding to the substrate is in competition with the lid strand preventing the closed conformation previously observed for the (*S*)-naringenin complex (PDB entry 4D06). The additional DQH binding in a cleft of two CHI hexamer units is promoted by crystal-packing effects and high DQH concentration during crystal-soaking experiments. This obviously weak binding may play a role in directing the substrate to the active site of CHI. We have observed a comparable situation with tetracyclines “waiting” at the entrance of the active site of tetracycline monooxygenase TetX [23].

With respect to the minor variations of the backbone atoms of the CHI monomers with and without occupied active sites, we obtain a typical lock-and-key mechanism with changes in side-chain conformations, only in the active site (His33, Glu91, and Phe93). Flexibility of the main chain is restricted to the lid.

After substrate binding, the lid-closure causes moderate active site side-chain movements, optimizing the activity by changing the permittivity (dielectric constant) of the active site. The lid is not essential for catalysis, because a Δ-lid variant showed five-fold reduced activity; which was still better than plant CHI [18].

## 4. Materials and Methods

### 4.1. Protein Preparation and Crystallisation

The protein expression, purification, and crystallization of CHI have been previously described in detail, including the cloning of the variant His33Ala [18].

In brief, crystallisation was performed with 6.8 mg/mL protein in 50 mM sodium phosphate buffer at pH 6.8. Crystals were obtained by the hanging-drop method using equal volumes of protein and reservoir solutions (2 μL) at 293 K. The reservoir solution consisted of ammonium sulphate, 0.2 M NaCl, and 0.1 M HEPES pH 7.5. The ammonium sulphate concentrations for native CHI and CHI_H33A were 1.8 M and 1.5 M, respectively. The crystals were soaked for 10 min in the reservoir solution with 2.5 mM (+)-taxifolin (2.5% ethanol) (2*R*,3*R*)-dihydroquercetin, (+)-taxifolin, Sigma-Aldrich, 78666, and HWI Analytik, 0389-05-85). The crystals were flash frozen in liquid nitrogen and stored until data collection. For all crystals, a cryoprotectant solution consisting of 22% glycerol, 1.8 M ammonium sulphate, 0.2 M NaCl, and 0.1 M HEPES pH 7.5 was used.

### 4.2. X-ray Data Collection Processing

In-house X-ray diffraction data of a (+)-taxifolin-soaked crystal of CHI_H33A were collected from a cryoprotected crystal at 100 K using a Saturn 92 CCD detector mounted on a MicroMax-007 rotating anode source (Rigaku MSC). The data were processed with the CrystalClear package 1.3.6 [24] (no cc_1/2_ given). The structure-factor amplitudes were calculated using TRUNCATE [25].

X-ray diffraction data of taxifolin soaked crystals of native CHI and CHI_H33A were collected at 100 K on beamline 14.1 at the BESSY II synchrotron, Berlin, Germany [26]. All diffraction images were processed with XDS [27] using the XDSapp graphical user interface [28]. Data collection and processing statistics are summarized in Table A1.

### 4.3. Structure Determination and Refinement

The crystallographic phase problems were solved by molecular replacement with the hexamer of CHI in complex with naringenin/naringenin chalcone (PDB entry 4D06) as the search model. Molecular replacement was carried out with Phaser [29]. The initially obtained models were improved and subsequently refined with REFMAC5 [30,31], including TLS segments and local non-crystallographic symmetry. Inspection of electron density maps and manual corrections of the models were performed with Coot [32]. The quality of the refined protein models was validated using MolProbity [33,34]. Figures were prepared with PyMOL [35]. Refinement statistics are listed in Table A2. The diffraction data and structural models were deposited into the Protein Data Bank.

## 5. Conclusions

The electron density maps of diffraction data of wild-type CHI crystals soaked with DQH do not provide the commonly preferred quality to describe ligand binding in a single static orientation. Instead, we see a static and/or dynamic disorder in the active site. This will be a challenging project in time-resolved crystallography, in order to take snapshots of the enzyme at work [36].

## Data Availability

X-ray diffraction data and atomic coordinates of the crystal structures of CHI/taxifolin, His33Ala/taxifolin, and His33Ala/taxifolin-overload have been deposited in the Protein Data Bank (PDB) with accession codes 8B7R, 8B7U, and 8B7Z, respectively.

## Appendix A

**Table A1:** Data collection statistics.

Protein–Ligand	CHI_H33A– Taxifolin-Overload	CHI_H33A– Taxifolin	CHI–Taxifolin
PDB entry	8B7Z	8B7U	8B7R
Radiation source	Rigaku MSC	BESSY II	BESSY II
beam line	MicroMax 007	BL 14.1	BL 14.1
Detector	Saturn 92 CCD	DECTRIS PILATUS 6M	DECTRIS PILATUS 6M
Wavelength (Å)	1.5418	0.9184	0.9184
Temperature (K)	100	100	100
unit cell parameters			
a (Å)	186.36	187.58	173.82
b (Å)	204.68	203.74	193.15
c (Å)	561.90	560.39	205.29
orthorhombic space group	*I*2_1_2_1_2_1_	*I*2_1_2_1_2_1_	*I*2_1_2_1_2_1_
Resolution range (highest shell) (Å) *	33.93–2.95	49.42–2.78	48.82–2.13
	(3.05–2.95)	(2.95–2.78)	(2.25–2.13)
Measured reflections	871370 (65227)	3660626 (562212)	1447977 (229327)
Unique reflections	222025 (21202)	266311 (42262)	191642 (30350)
Averaged Redundancy	3.92 (3.06)	13.7 (13.3)	7.56 (7.56)
Completeness (%)	98.8 (95.1)	99.3 (98.2)	99.5 (98.3)
Rmeas (%)	28.9 (76)	36.8 (158)	18.8 (110)
Mean *I*/σ(*I*)	4.1 (1.0)	9.3 (1.76)	10.74 (1.93)
CC ½		0.99 (0.24)	1.0 (0.33)
Wilson *B*-factor (Å^2^)	47.3	42.4	36.5

* Values in parentheses correspond to the highest resolution shell.

**Table A2:** Refinement statistics [30,31,33,35].

Protein–Ligand	CHI_H33A– Taxifolin-Overload	CHI_H33A– Taxifolin	CHI–Taxifolin
PDB entry	8B7Z	8B7U	8B7R
Resolution range	33.95–3.00	49.42–2.80	48.82–2.15
Rcryst (%)/number of reflections	24.9/206779	21.3/257795	16.3/183628
Rfree (%)/number of reflections	26.8/4272	22.4/2736	19.0/2035
Number of non-hydrogen atoms: protein/solvent/ligands	39253 all 38066/45/1142	39179 all 38164/233/782	14852 all 12890/1830/132
RMSD * bond lengths (Å)	0.009	0.007	0.011
RMSD bond angles (°)	1.50	1.33	1.54
RMSD torsion angles (°)	7.77	8.01	7.73
Ramachandran parameters (%) favoured/allowed/outlier			
	88.1/11.8/–	87.5/12.5/–	90.4/9.6/–
Average B-factors (Å^2^)	58.1	66.9	35.3
Matthews coefficient V_M_ (A^3^ Da^−1^)	4.88	4.88	4.14
Corresponding solvent content (%)	75	75	70

* RMSD, root mean square deviation.
